# Letter to the editor from Miller et al: “long-acting growth hormone for treating growth hormone deficiency in children: a meta-analysis of randomized controlled trials focusing on changes in body mass index”

**DOI:** 10.1210/clinem/dgag158

**Published:** 2026-04-11

**Authors:** Bradley S Miller, Lars Sävendahl, Michael Højby, Michel Polak

**Affiliations:** Division of Endocrinology, Department of Pediatrics, University of Minnesota Medical School, M Health Fairview Masonic Children's Hospital, Minneapolis, MN 55454, USA; Division of Pediatric Endocrinology, Karolinska University Hospital, 171 64 Solna, Sweden; Department of Women's and Children's Health, Karolinska Institutet, SE-171 77 Stockholm, Sweden; Medical & Translational Science, Novo Nordisk A/S, 2860 Søborg, Denmark; Service D'Endocrinologie, Gynécologie et Diabétologie Pédiatriques, Hôpital Universitaire Necker Enfants Malades Paris, Université Paris Cité Assistance Publique-Hôpitaux de Paris, Paris 75015, France

To the Editor,

Levaillant et al conducted a meta-analysis to investigate change in body mass index (BMI) with three different long-acting growth hormones (LAGHs) (lonapegsomatropin, somapacitan, and somatrogon) compared with daily growth hormone (GH) in children with GH deficiency (GHD) (1). The authors concluded that LAGH treatment is associated with significant increase in BMI standard deviation score (SDS) during the first year, compared with daily GH. In extension phases, significant increase in BMI SDS was observed in children switching from daily GH to LAGH, whereas it remained stable in those continuing LAGH. Height SDS results were similar between LAGH and daily GH. These findings were similar across the different LAGH molecules.

Data from one of the largest real-world evidence registries have previously shown that daily GH treatment is associated with net increase in BMI SDS between treatment start and near adult height achievement (2). As noted by the authors, BMI is a surrogate measure and does not provide information on body composition, ie, fat or lean body mass. However, body composition analyses and appropriate imaging measures are very difficult to conduct and interpret in prepubertal children, particularly without segmenting those at higher risk of weight gain (for example, those with multipituitary GHD or comorbidities) and data remain limited. In the adult GHD context, fat mass reductions and lean body mass increases have been observed during treatment with daily GH and LAGH, indicating improvement in body composition (3).

Based on 7-year data from REAL 3 (4) and 3-year data from REAL 4 (5), BMI SDS remained within the normal range (±2 SDS) in patients treated with somapacitan, and similar results have been observed with lonapegsomatropin after 6 years in enliGHten (6). These results suggest the benefit-risk profile of LAGHs in children with GHD is favorable. The BMI increase associated with the first year of LAGH treatment, as reported for lonapegsomatropin, somapacitan, and somatrogon, is modest and nonprogressive, stabilizing after 1 year, and no further increases or impacts on metabolic parameters have been observed. In our view, the observed moderate BMI SDS increase does not warrant changes to clinical practice recommendations.

Levaillant et al highlighted that family-based interventions are associated with improved body composition in growing children; however, the impact of lifestyle factors was not evaluated in the studies included in the meta-analysis. Lifestyle interventions may not be necessary since the BMI increase may be due to lean mass increase. Routine clinical practice should continue to include consideration of lifestyle factors, longitudinal growth monitoring, BMI, and metabolic parameters. Similarly, the meta-analysis conducted by Levaillant et al did not account for differences in IGF-I profiles between the 3 different LAGH molecules, which is a methodological limitation.

To date, there are limited data on body composition in pediatric patients with GHD. Further studies are warranted to evaluate the mechanisms behind the modest BMI increase associated with LAGH during the first year of treatment; specifically, the short- and long-term effects on fat and lean body mass should be explored by appropriate body composition imaging measures.
